# Specific detection of dengue and Zika virus antibodies using envelope proteins with mutations in the conserved fusion loop

**DOI:** 10.1038/emi.2017.87

**Published:** 2017-11-08

**Authors:** Alexandra Rockstroh, Beyene Moges, Luisa Barzon, Alessandro Sinigaglia, Giorgio Palù, Widuranga Kumbukgolla, Jonas Schmidt-Chanasit, Manoel Sarno, Carlos Brites, Andres Moreira-Soto, Jan Felix Drexler, Orlando C Ferreira, Sebastian Ulbert

**Affiliations:** 1 Department of Immunology, Fraunhofer Institute for Cell Therapy and Immunology, Perlickstrasse 1, Leipzig 04103, Germany; 2 Department of Molecular Medicine, University of Padova, via A. Gabelli 63, Padova 35121, Italy; 3 Faculty of Medicine and Allied Sciences, Rajarata University of Sri Lanka, Mihinthale 50008, Sri Lanka; 4 WHO Collaborating Centre for Arbovirus and Haemorrhagic Fever Reference and Research, Bernhard Nocht Institute for Tropical Medicine, Bernhard Nocht Strasse 74, Hamburg 20359, Germany; 5 German Centre for Infection Research (DZIF); 6 Hospital Universitário Professor Edgard Santos, Universidade Federal de Bahia, Salvador 40110-060, Brazil; 7 Maternidade Climério de Oliveira, Universidade Federal da Bahia, Salvador 40005-150, Brazil; 8 Institute of Virology, University of Bonn Medical Centre, Bonn 53127, Germany; 9 Charité—Universitätsmedizin Berlin, corporate member of Freie Universität Berlin, Humboldt-Universitätzu Berlin, and Berlin Institute of Health, Institute of Virology, Berlin 10117, Germany; 10 Laboratory of Molecular Virology, Departamento de Genética, Universidade Federal do Rio de Janeiro (UFRJ), Rio de Janeiro 21941-901, Brazil

**Keywords:** dengue virus, Zika virus, diagnosis, cross-reactivity, ELISA, envelope proteins

## Abstract

Detection of antibodies is widely used for the diagnosis of infections with arthropod-borne flaviviruses including dengue (DENV) and Zika virus (ZIKV). Due to the emergence of ZIKV in areas endemic for DENV, massive co-circulation is observed and methods to specifically diagnose these infections and differentiate them from each other are mandatory. However, serological assays for flaviviruses in general, and for DENV and ZIKV in particular, are compromised by the high degree of similarities in their proteins which can lead to cross-reacting antibodies and false-positive test results. Cross-reacting flavivirus antibodies mainly target the highly conserved fusion loop (FL) domain in the viral envelope (E-) protein, and we and others have shown previously that recombinant E-proteins bearing FL-mutations strongly reduce cross-reactivity. Here we investigate whether such mutant E-proteins can be used to specifically detect antibodies against DENV and ZIKV in an ELISA-format. IgM antibodies against DENV and ZIKV virus were detected with 100% and 94.2% specificity and 90.7% and 87.5% sensitivity, respectively. For IgG the mutant E-proteins showed cross-reactivity, which was overcome by pre-incubation of the sera with the heterologous antigen. This resulted in specificities of 97.1% and 97.9% and in sensitivities of 100% and 100% for the DENV and ZIKV antigens, respectively. Our results suggest that E-proteins bearing mutations in the FL-domain have a high potential for the development of serological DENV and ZIKV tests with high specificity.

## INTRODUCTION

Arthropod-transmitted flaviviruses are small, enveloped RNA viruses, which are endemic to many parts of the world. They include a large number of important human pathogens, such as dengue, Zika, yellow fever, West Nile, Japanese encephalitis and tick-borne encephalitis viruses (DENV, ZIKV, YFV, WNV, JEV and TBEV, respectively).^1^ On the basis of their antigenic properties, flaviviruses are divided into distinct serocomplexes, such as the JEV serocomplex (which contains JEV, WNV and others), or the DENV serocomplex (with the different dengue virus serotypes).^2^ Among flaviviruses, DENV is causing the most severe impact on human health. Current estimates for DENV infections reach 400 million worldwide, occurring in over a hundred tropical and subtropical countries, and leading to thousands of deaths per year.^3^ Recently, several candidate DENV-vaccines have been tested in clinical trials, and the first product was licensed in some endemic countries.^4, 5^ ZIKV, which had remained unnoticed in Africa for decades, emerged in the South Pacific in 2007 and was introduced to South America in 2014, where it is currently spreading.^6, 7^ ZIKV causes febrile illness, but it also appears to be linked to Guillain-Barré syndrome as well as microcephaly in newborns.^8^ Many of the human pathogenic flaviviruses are transmitted by the same mosquito species (especially of the genus *Aedes*), and areas where different flaviviruses co-circulate are increasing in number, most importantly DENV and ZIKV in South America.^9, 10^

Serological diagnosis of flavivirus infections relies on the measurement of IgM and IgG antibodies which usually appear about a week after symptoms onset.^11, 12, 13^ Acute infections are diagnosed either by the detection of IgM or by a rising IgG antibody titer from the acute to the convalescent phases of infection. In secondary flavivirus infections, a measurable IgM response can be very low or absent ^7, 14^ and IgG tests become the method of choice. However, specificity in flavivirus antibody-measurement is significantly hampered by the structural similarity of the viruses and the resulting antibody cross-reactivity.^15, 16, 17^ This is of considerable concern especially for IgG-based assays, as it leads to false-positive test results.^18, 19^ The massive co-circulation of ZIKV and DENV has dramatically increased the problem. Antibodies against ZIKV are particularly cross-reactive to DENV and vice versa due to conserved parts of their proteins.^2^ As a consequence, currently available tests are severely compromised by cross-reacting antibodies.^20^ To validate serological results, virus neutralization tests have to be performed, which are time consuming and require BSL-3 facilities.^21^ Therefore, there is a strong need for the development of techniques that have high sensitivity and specificity to detect and differentiate flavivirus antibodies and allow a high-throughput analysis.

The predominant target for cross-reacting antibodies is the fusion loop (FL) domain in the flavivirus envelope (E) protein, a short amino-acid sequence which is almost identical in many pathogenic flaviviruses.^22, 23, 24^ It was shown before that mutating residues within this domain reduces binding of cross-reactive antibodies.^25, 26, 27^ By using recombinant proteins that contain point mutations within and near the FL, we have previously generated systems to specifically diagnose WNV and DENV infections, respectively.^28, 29^ Here, we have analyzed the potential of this technology to differentiate antibody responses to DENV and ZIKV infections. Our results suggest that FL-mutant E-proteins of ZIKV and DENV can be used for a specific serological diagnosis of both infections.

## MATERIAL AND METHODS

### Human serum samples

Serum samples were divided into different groups regarding their virus infections, described in Tables 1 and 2. The origins of the samples were as follows:

DENV (*n*=15), ZIKV (*n*=14) and CHIKV (*n*=8) IgM and/or IgG-positive samples were obtained from persons returning from a stay in endemic areas, TBEV seropositive samples (*n*=24) were obtained from a seroprevalence study in forest rangers in Northeastern Italy, WNV seropositive sera (*n*=28) were obtained from patients with neuroinvasive disease or fever during outbreaks in Northeastern Italy. All these samples, as well as 14 negative control sera, were collected and characterized at Padova University Hospital, Italy. Ethical approval for these studies was obtained from the Padova University Hospital Ethics Committee. Another DENV (*n*=31), ZIKV (*n*=18) and JEV (*n*=4) IgM and/or IgG positive serum samples were obtained from returning travelers after a stay in endemic areas and were collected at the Bernhard Nocht Institute for Tropical Medicine, Hamburg, for diagnostic purposes with written informed consent from each patient. DENV PCR positive sera (*n*=42) were collected in Sri Lanka with the approval of Ethical Review Boards of the Medical Research Institute and Lady Ridgway Children Hospital Colombo. 13 ZIKV IgG positive sera were collected in Salvador, Brazil, from mothers having children with ZIKV infection-associated microcephaly. Sampling and testing were approved by the institutional research ethics board of the federal university of Bahia Climério de Oliveira. Another DENV (*n*=14) IgG and ZIKV (*n*=9) IgM and/or IgG positive samples were collected in Rio de Janeiro, Brazil, with ethical approval by the ethical committee from the University Hospital Clementino Fraga Filho (HUCFF). Additional DENV-positive samples (*n*=14), as well as one negative control, were obtained from Sera Care Life Sciences (Milford, CT, USA) and from Zeptometrix Corporation (Buffalo, NY, USA) (4 DENV-positive samples and two negative controls) with sample origin from Colombia, Honduras and Ecuador. The YFV (*n*=8) samples (obtained from the Robert Koch Institute, Berlin, Germany) were from YFV-vaccinated individuals who had participated in a randomized controlled vaccination study (approved by the national ethics committee). The Malaria immune sera (*n*=6) from Ghana were obtained from the Fraunhofer Institute for Molecular Biology and Applied Ecology, Aachen, Germany. Ethical clearance was obtained from the Committee on Human Research Publication and Ethics of the Kwame Nkrumah University of Science and Technology. All participants in this study provided informed consent and all samples were analyzed anonymously.

### Antigens

The quadruple mutant E-protein from ZIKV (strain H/PF/2013, E-protein amino acid residues 1–406) bearing the point mutations T76A, Q77G, W101R and L107R was cloned into pMT/BiP/V5-His vector (Invitrogen, Carlsbad, CA, USA), expressed in *Drosophila S2* cells and purified from cell culture supernatants with IMAC and size exclusion chromatography as previously described for the DENV quadruple mutants,^29^ which were generated accordingly. For serological IgM and IgG assays, the four DENV 1–4 mutant antigens were mixed in ratios of 1:1:1:1 and 1:1:1:0.2 (due to increased cross-reactivity of DENV 4 Equad protein in IgG measurements, as described ^29^), in concentrations of 300 ng and 160 ng per well, respectively, as described.^29^ The ZIKV Equad antigen was used in the indicated amounts (Results section).

### Antibody measurements

Indicated amounts of ZIKV Equad or DENV 1–4 Equad mixtures were coated overnight on Nunc polysorb plates (Thermo Scientific) in 100 μL coating buffer (15 mM Na2CO3, 35 mM NaHCO3, pH 9.6) at 4 °C. The plates were washed three times with 350 μL per well of PBS-0,05% Tween and blocked with 200 μL of 5% non-fat milk powder (blocking solution) for 2 h at room temperature. After a second washing step, human sera were diluted 1:100 in 100 μL blocking solution per well and incubated for 1.5 h at room temperature. Following a third washing step, the HRP-conjugated secondary goat anti human IgG (BioRad, Hercules, CA, USA, 1:10 000 in 100 μL blocking solution per well) or rabbit anti human μ-chain IgM (Dianova, 1:5000 in 100 μL blocking solution per well) antibody was added for 1 h at room temperature. After a fourth washing step, 100 μL TMB substrate (Biozol) per well were incubated for 30 min at room temperature. The reaction was stopped with 50 μL 1 M H_2_SO_4_ and signals were read out at 450 nm with background reduction at 520 nm in a micro plate reader (Infinite M200, Tecan).

In competition IgG ELISAs, sera (diluted 1:100 in blocking solution) were pre-incubated with indicated amounts of the competing antigen for 1 h at room temperature. Subsequently, they were added to the blocked antigens on the ELISA plate and incubated for 1.5 h at room temperature. The protocol was then continued as described above with IgG antibody detection.

### Statistical analysis

All antibody measurements were performed in duplicates in at least two independent experiments, except in Figure 6, where single measurements were performed due to limited amounts of serum. Graphical and descriptive statistical analysis of data was carried out using GraphPad Prism 6 (La Jolla, CA, USA). Statistical significance was determined using the Holm-Sidak method, with alpha=5.000%. Receiver operating characteristics (ROC) optimal curve calculations were performed in GraphPad Prism 6 with ELISA signals of the infected specimen and negative sera as control values. Signal cutoffs with optimal sensitivity and specificity were chosen and data were interpreted as positive with a signal/cutoff ratio higher than1.1 to ensure the best specificity. The cutoffs for the individual assay types are listed in Supplementary Table S1.

## RESULTS

To facilitate a specific serological differentiation between DENV and ZIKV infections, we inserted four amino acid point mutations in the conserved fusion loop (FL) domain of the ZIKV E-protein (Equad) and compared it to the previously described DENV 1–4 Equad mixture, which was shown to significantly reduce cross-reactivities in dengue serological diagnosis.^29^

**Figure 1:**
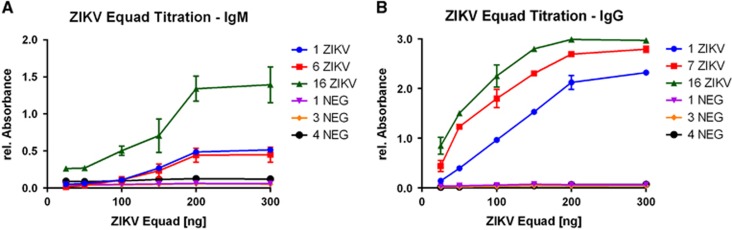
Titration curves of ZIKV Equad in an (**A**) IgM and (**B**) IgG ELISA with ZIKV- positive and negative sera.

The optimal concentration of ZIKV Equad for ELISA-based IgG and IgM tests was established through titration of the antigen with three ZIKV-positive and -negative sera each (Figure 1). Saturation of ZIKV-positive signals was observed at 200 ng per well in IgM- and IgG-measurements. Negative sera did not show any background in both setups through all tested antigen amounts, indicating a high specificity of the purified ZIKV Equad.

For the DENV 1–4 Equad mixture, the antigen amount per well yielding optimal sensitivity and specificity in IgM- and IgG- based ELISAs was determined previously.^29^

**Figure 2:**
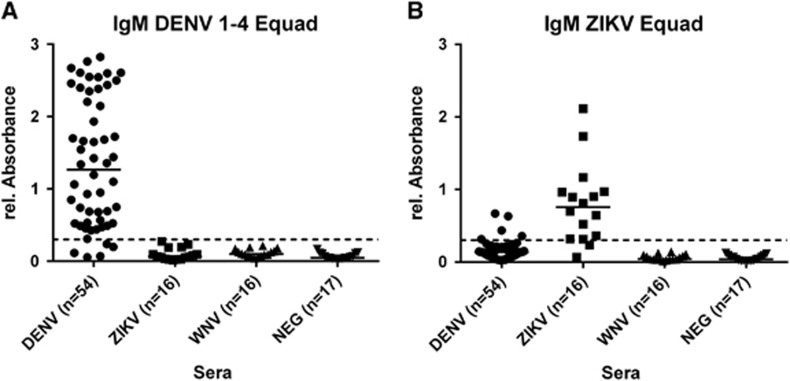
IgM ELISA on (**A**) 300 ng per well of DENV 1–4 Equad and (**B**) 200 ng per well of ZIKV Equad tested with IgM-positive DENV, ZIKV, WNV and flavivirus negative human sera. One sample per patient was examined in two independent experiments and plotted as a mean data point indicating absorbance values. The dotted lines represent cutoffs determined by a ROC analysis with negative sera as controls.

IgM antibodies of DENV-, ZIKV- and WNV-infected human sera (Table 1) and negative control sera were measured on the DENV (1–4) Equad mixture (Figure 2A) and on ZIKV Equad (Figure 2B). The sera from DENV- and ZIKV- infected individuals showed specific binding on DENV 1–4 Equad and ZIKV Equad, respectively. Negative control sera were used to calculate a ROC cutoff for each antigen (Supplementary Table S1) resulting in 90.7% sensitivity for DENV Equad and 87.5% sensitivity for ZIKV Equad. Both antigens showed 100% specificity of infected sera in comparison to the flavivirus-negative control samples (Table 3, Supplementary Table S2). No cross-reactivity was observed on DENV 1–4 Equad with ZIKV- and WNV-IgM-positive sera, resulting in 100% specificity of this assay (Figure 2A). The ZIKV Equad antigen yielded 5/54 cross-reactive DENV- infected samples reducing its specificity versus DENV to 90.7%(total specificity of 94.3% with all ZIKV-negative control samples). A statistical analysis of all measurements described is shown in Supplementary Tables S2–S4.

**Figure 3:**
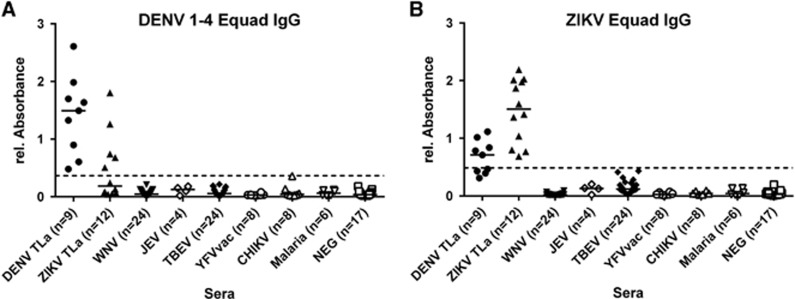
IgG ELISA on (**A**) 160 ng per well of DENV 1–4 Equad and (**B**) 150 ng per well of ZIKV Equad measured with flavivirus positive sera, CHIKV and Malaria positive and negative sera. One sample per patient was examined in two independent experiments and plotted as a mean data point indicating absorbance values. The dashed lines represent cut-offs determined by a ROC analysis with negative sera as controls.

Next, we used the DENV 1–4 Equad mixture and ZIKV Equad to measure IgG responses in serum samples from DENV-, ZIKV-, WNV-, JEV- and TBEV-infected persons (Figure 3). For DENV and ZIKV, sera derived from European travelers returning from endemic areas (groups DENV-TLa, ZIKV-TLa, Table 2) were used, which represent mostly primary infections. Non-infected individuals as well as samples from CHIKV- and Malaria-infected and YFV-vaccinated persons served as controls. Sera of non-infected individuals were used for ROC analysis and cutoff calculation in comparison to the DENV- and ZIKV-positive serum samples resulting in 100% sensitivity for both antigens (Table 3, Supplementary Table S1). Sera from WNV-, JEV-, TBEV-, CHIKV-, Malaria-infected and YF-vaccinated individuals showed no cross-reactivity on the DENV- and ZIKV- Equad antigens and were detected as negative according to the ROC cutoffs. For DENV- and ZIKV- positive sera, a statistically significant overall signal reduction was observed when DENV-positive specimen were measured on ZIKV Equad and ZIKV-positive sera on the DENV Equad in comparison to signals on the homologous antigen (Supplementary Table S3). However, when using the ROC cutoff, both groups displayed cross-reactive signals leading to several false-positive results of ZIKV-infected individuals in the DENV- IgG-ELISA and of DENV-infected individuals in the ZIKV-IgG-ELISA.

**Figure 4:**
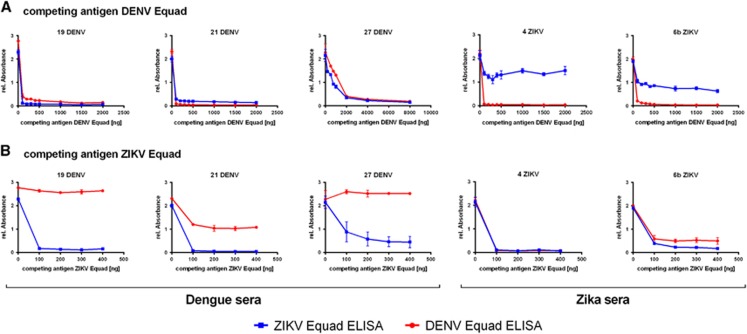
Titration of the competing antigen (**A**) DENV Equad and (**B**) ZIKV Equad in Dengue and Zika positive sera and IgG measurement on the coated DENV Equad (blue lines) and ZIKV Equad (red lines).

To further reduce the IgG cross-reactivity between DENV- and ZIKV- positive sera, a competition ELISA setup was chosen and tested with 3 DENV-positive sera which were known to be ZIKV-negative because they were collected in South America before the ZIKV-outbreak and 2 ZIKV-infected samples. All of them displayed similarly high signals in both the DENV- and the ZIKV- IgG ELISA. These sera were now pre-incubated with a competing antigen (either ZIKV Equad or the DENV 1–4 Equad mixture) and then measured on the coated DENV 1–4 Equad or ZIKV Equad. Figure 4A displays competition experiments with DENV 1–4 Equad that resulted in a signal drop for dengue positive sera on both antigens caused by the removal of DENV-specific and ZIKV-cross-reactive antibodies with increasing amounts of DENV 1–4 Equad as competing antigen. In the DENV-positive serum 27, removal of DENV- and cross-reactive ZIKV-antibodies was seen only with 2 μg competitor antigen, about 10fold more than in the other samples. Competition of ZIKV-positive sera with DENV 1–4 Equad also strongly decreased the signals in DENV-ELISAs, but only slightly influenced the signals on ZIKV Equad through all tested competing antigen amounts. The competition with at least 200 ng of ZIKV Equad (Figure 4B) eliminated ZIKV cross-reactive antibodies in DENV-infected sera, which then showed low signals in ZIKV-ELISAs and still well detectable signal intensities in DENV-ELISAs. For one specimen (21DENV) a signal drop was observed in DENV-ELISA with ZIKV-competition (Figure 4B), similar to the ZIKV-infected samples with DENV-competition in the ZIKV-ELISA (Figure 4A). This illustrates the varying amounts of cross-reactive antibodies in individual serum samples. The two ZIKV-positive sera that were competed with ZIKV Equad showed a signal drop with 200 ng competitor in both ZIKV- and DENV-ELISAs (Figure 4B).

**Figure 5:**
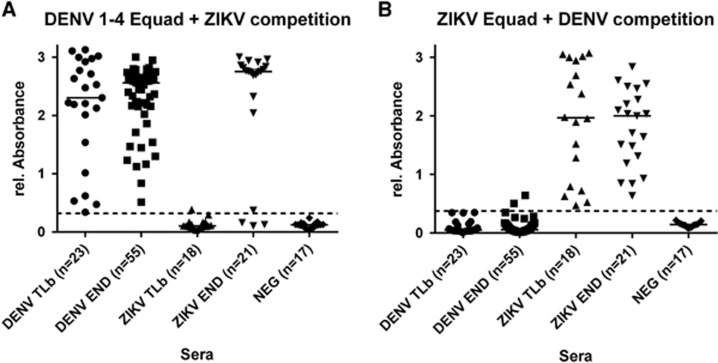
IgG Competition ELISA: Sera were measured on (**A**) 160 ng/well of DENV 1–4 Equad with 200 ng per well ZIKV Equad competition and (**B**) 150 ng of ZIKV Equad with 2 μg of DENV 1–4 competition. One sample per patient was examined in two independent experiments and plotted as a mean data point. The dashed lines represent cutoffs determined by a ROC analysis with negative sera as controls.

After having established suitable conditions for competition experiments, DENV- and ZIKV- IgG-positive as well as the negative sera were then measured on DENV 1–4 Equad after competition with 0.2 μg ZIKV Equad (Figure 5A) and on ZIKV Equad after competition with 2 μg of DENV 1–4 Equad (Figure 5B). The higher amount of the DENV antigen as a competitor was chosen in order to correctly analyze sera such as sample 27DENV (Figure 4A), which only showed effective competition after incubation with 2 μg of DENV 1–4 Equad.

**Figure 6:**
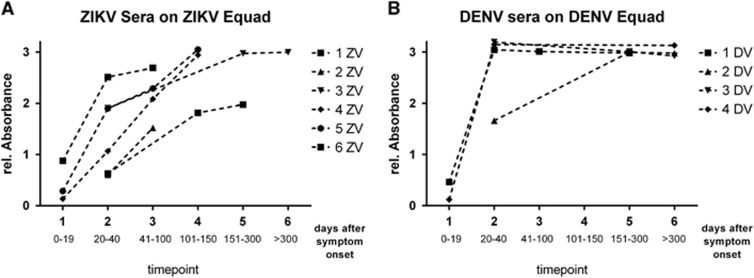
IgG competition ELISA of paired serum samples from European travelers infected with ZIKV (**A**) and DENV (**B**) measured on 150 ng of ZIKV Equad (**A**) with DENV Equad competition and on 160 ng of DENV 1–4 Equad (**B**) with ZIKV competition.

ZIKV- and DENV-positive sera were divided into two different groups each (Table 2): European travelers returning from endemic countries (primary infections, DENV-TLb and ZIKV-TLb) and patients from DENV endemic areas in Brazil and Sri Lanka (DENV-END). A co-infection with ZIKV in the DENV-END group could be excluded because Brazilian samples were taken in 2008, before the ZIKV outbreak, and Sri Lanka samples were from 2015 with no known circulation of ZIKV. However, secondary DENV-infections were included in this group. In contrast, the samples in the ZIKV-END group all derived from DENV-endemic areas in Brazil and were taken in 2016. A previous DENV-infection is therefore likely and was also found previously with serological test methods in 90% of those samples (Table 2). In comparison to the IgG assay without competition, the specificity of both the DENV and the ZIKV Equad ELISAs (Figure 5 and Supplementary Table S4) was strongly enhanced (100% for ZIKV Equad and 94.4% for DENV Equad) in the DENV-TLb and ZIKV-TLb groups. Also in the DENV-END group, all serum samples were detected as positive for DENV and only two signals were over the cutoff on the ZIKV antigen. The ZIKV-END samples were all detected as positive for ZIKV (100% sensitivity), but 85.7% of the samples also reacted in the DENV-ELISA, which correlates well to the prior DENV infections present in this group. The course of IgG production against ZIKV and DENV was also determined using paired samples from the returning traveler groups. As can be seen from Figure 6, antibody levels increased during the acute phase in the first 40 days after symptom onset and remained stable for up to 300 days.

## DISCUSSION

Cross-reactivity is a long-known complication in the serological diagnosis of flavivirus-infections, and several attempts have been made to develop systems to increase specificities of available test systems.^25, 26, 28, 29, 30^ However, due to the epidemic spread of ZIKV in areas of simultaneous DENV circulation, the problem has gained another dimension. The specific diagnosis of these infections is mandatory not only for the implementation of effective control and surveillance activities, including the conduction of recently started DENV vaccine trials, but also for the timely treatment of potentially life-threatening disease symptoms. However, available DENV-tests are severely affected by ZIKV-antibodies, leading to a large number of false positive test results.^20, 31, 32^ Recently, ZIKV ELISAs based on NS1-proteins as antigens have been made commercially available, but limitations in IgM and/or IgG detection have been reported.^33, 34, 35, 36^ Here we present a system for the specific and sensitive serological diagnosis of DENV- and ZIKV infections, based on the E-protein as antigen. The E-protein is widely used in serological flavivirus diagnosis, as virtually all infected individuals generate antibodies against it. On the other hand, it is targeted by cross-reactive antibodies, which mainly recognize the conserved FL-domain. By using mutations within and next to the FL-domain of the E-proteins of DENV and ZIKV, we found that IgM antibodies show greatly reduced cross-reactivity and bind specifically to the homologous antigen. This is in line with previous reports indicating that IgM responses against flaviviruses are more specific than IgG,^19^ although cross-reactivity has also been described.^7, 37^ However, IgM antibodies are often produced only in low amounts, especially in secondary flavivirus infections or after a vaccination and are shortlived,^14, 38^ hence IgG measurements become necessary. Using the mutant E-proteins, IgG antibodies present in the DENV- and ZIKV-positive sera did show cross-reactive binding to the ZIKV- and the DENV-antigens, respectively, although serum samples from WNV-, JEV- and TBEV-infected persons remained negative in both ELISAs, confirming previous results with the DENV antigen.^29^ On the basis of the sequence conservation of the E-protein ectodomains, ZIKV is more closely related to DENV than to the JEV-serocomplex, with amino acids identities of 52% and 54–57% when comparing ZIKV to WNV and DENV (four serotypes), respectively.^2,39^ This minor difference in amino acids identities is apparently enough to result in a very different binding of cross-reactive antibodies in sera from infected persons. Antibodies against non-flaviviral vector-borne pathogens (malaria, chikungunya) did not bind to either antigen, which underlines the usability of the mutant E-proteins in the diagnosis of flavivirus infections, whereas false-positive results with sera from malaria infected persons have been reported for an NS1-based ZIKV assay.^40, 41^

In order to increase the specificity of the test in the differentiation of DENV- from ZIKV IgG-antibodies and vice versa, we performed competition experiments by pre-incubating the sera with one antigen before measuring them on the ELISA plate coated with the second one. This resulted in a statistically significant reduction of cross-reactivities (Supplementary Table S3). Now, ZIKV-infected samples from returning travelers did not show signals in the DENV ELISA, and the same was the case for DENV-infected returning travelers in the ZIKV-test. This demonstrates that, after the competition step, the mutant antigens are able to differentiate DENV- and ZIKV- IgG responses. When analyzing DENV-positive samples from inhabitants of DENV-endemic areas, these were detected as DENV-positive with high specificity and sensitivity and only 2/55 displayed low signals over the cutoff in the ZIKV ELISA. Whereas DENV secondary infections can be assumed for many of the samples within this group, ZIKV-infections can be excluded. The ZIKV-positive serum samples in the ZIKV-END group derived from areas with a high DENV-seroprevalence that can reach up to 80%,^42^ which corresponds to the detection of DENV-antibodies in 18/21 samples using the mutant antigens. This result underlines the need to simultaneously test for ZIKV- and DENV- IgG antibodies in areas of co-circulation.

Such secondary flavivirus infections represent a major challenge, not only for serological diagnosis. Pre-existing flavivirus antibodies might lead to severe complications of acute DENV- or ZIKV-infections, including severe dengue and neurological or congenital complications with ZIKV, as has been suggested by several studies.^43, 44, 45^ Therefore, tests to identify and discriminate such antibodies are an unmet need which has to be addressed urgently. Methods as the one presented here, which are able to detect DENV and ZIKV IgM- and IgG- antibodies with high specificity and sensitivity, could be very useful in this respect.

Using paired serum samples we analyzed the rise of IgG antibodies upon infection with ZIKV and DENV in returning European travelers. A strong increase in the first 40 days was observed, and DENV-antibodies seemed to reach a plateau earlier than ZIKV antibodies, which might be attributed to a generally higher viral load in infections with DENV compared to ZIKV.^46^ However, higher numbers of serum samples need to be analyzed in order to test whether this is indeed a difference between the two infections

Most of the currently available ZIKV-ELISAs are based on the NS1 protein. Recently published attempts for improvement of ZIKV serological diagnosis include a microarray-based assay using ZIKV NS1 and DENV virus particles in a multiplexed format based on a plasmonic gold platform.^47^ and a monoclonal ZIKV NS1-antibody in a blockage-of-binding format.^48^ The system presented here relies on another antigen (mutant E-protein) and could, therefore, be used as an alternative approach in serology, independent of NS1 and also not dependent on only a single epitope. As the infrastructure for virus neutralization tests is only available in few specialized laboratories, confirmation and evaluation of inconclusive results from a particular serological assay can be performed best with an assay that is based on a different antigen. Therefore, resulting ELISAs for ZIKV and DENV could contribute to an accurate diagnosis and surveillance of these two virus infections, both as stand-alone tests and as means to complement existing methods.

## Figures and Tables

**Table 1:** Description of serum groups used for IgM measurements

**Group**	***N***	**Characteristics**	***N***	**Origin**	**Year of sample collection**	**Diagnosis**			
						**PCR**	**Neutralization test**	**IgM ELISA**	**IgG ELISA**
**DENV**	54	Acute sera from returning travelers and DENV endemic regions	12	Italy	2013–2016	10	4	12	7
			2	Zeptometrix		0	2	2	2
			4	Seracare (Colombia, Honduras, Equador)	2011	0	4	4	4
			17	Sri Lanka	2015	17	0	17	n.t.
			19	Germany	2011–2016	14	0	19	18
**ZIKV**	16	Acute sera from returning travelers and DENV endemic regions	2	Brazil	2015–2016	1	1	2	2
			8	Italy	2015–2016	6	2	8	7
			6	Germany	2016–2017	4	1	6	6
**WNV**	16	Residents in WNV endemic regions	16	Italy	2012–2013	0	16	16	16

Abbreviation: not tested, n.t.

Diagnosis was performed at the laboratories supplying the samples for this study, using commercial and in house assays.

**Table 2:** Description of serum groups used for IgG measurements

**Group**	**Characteristics**	**Subgroup**	***N***	**Origin**	**Year of sample collection**	**Diagnosis**			
						**PCR**	**Neutralization test**	**IgM ELISA**	**IgG ELISA**
**DENV TL**	Returning travelers	DENV TLa (Figure 3)	8	Italy	2013	4	6	5	8
		DENV TLb (Figure 5)	22	DENV TLa and Germany (*n*=14)	2011–2016	12	6	11	21
**DENV END**	Residents in DENV endemic regions	—	55	Zeptometrix (*n*=2)	2013	0	2	0	2
				Seracare (Colombia, Honduras, Equador) (*n*=14)	2011	0	14	4	14
				Brazil (*n*=14)	2008	0	8	0	14
				Sri Lanka (*n*=25)	2015	25	n.t.	n.t.	25
**ZIKV TL**	Returning travelers	ZIKV TLa (Figure 3)	12	Italy	2016	6	6	4	12
		ZIKV TLb (Figure 5)	19	ZIKV TLa and Germany (*n*=7)	2016–2017	9	7	4	19
**ZIKV END**	Residents in DENV endemic regions	—	21	Brazil	2015–2016	6	14	1	21 (19 also positive for DENV IgG)
**WNV**	Residents in WNV endemic regions	—	24	Italy	2012–2013	2	23	15	24
**JEV**	Returning travelers	—	4	Germany	2013–2016	0	n.t.	4	4
**TBEV**	Residents in TBEV endemic region	—	24	Italy	2013	0	n.t.	0	24
**CHIKV**	Returning travelers	—	8	Italy and Germany	2014–2015	0	0	7	8
**YFV vac**		—	8	Germany	2011–2013	n.t.	8	n.t.	8
**Malaria**		—	6	Ghana	2012	n.t.	n..t	n.t.	6

Abbreviation: not tested, n.t.

Diagnosis was performed at the laboratories supplying the samples for this study, using commercial and in house assays; n.t.=not tested.

**Table 3:** Sensitivity and specificity of DENV- and ZIKV- Equad in IgM- and IgG- measurements

**DENV Equad**						**ZIKV Equad**					
**Sera**	**N**	**Positive**	**Sensitivity**	**Specificity**	**95% CI**	**Sera**	**N**	**Positive**	**Sensitivity**	**Specificity**	**95% CI**
*IgM*											
**DENV**	**54**	**49**	90.74%	—	79.70%–96.92%	DENV	54	5		90.74%	79.70%–96.92%
ZIKV	16	0	—	100%	79.41%–100%	**ZIKV**	**16**	**14**	87.5%	—	61.65%–98.45%
WNV	16	0	—	100%	79.41%–100%	WNV	16	0		100%	79.41%–100%
NEG	17	0	—	100%	80.49%–100%	NEG	17	0		100%	80.49%–100%
Total control sera	49	0		**100.00**	92.75%–100%	Total control sera	87	5		**94.25%**	84.12%–96.70%

*IgG*											
**DENV Tla**	**9**	**9**	100%	—	63.37%–100%	DENV TLa	9	5	—	44.44%	10.70%–48.41%
ZIKV Tla	12	5	—	58.33%	18.44%–67.08%	**ZIKV TLa**	**12**	**12**	100%	—	15.70%–84.30%
WNV	24	0	—	100%	85.75%–100%	WNV	24	0		100%	85.75%–100%
JEV	4	0		100%	39.76%–100%	JEV	4	0		100%	39.76%–100%
TBEV	24	0	—	100%	85.75%–100%	TBEV	24	0		100%	85.75%–100%
CHIKV	8	0	—	100%	63.06%–100%	CHIKV	8	0		100%	63.06%–100%
YFVvac	8	0	—	100%	63.06%–100%	YFVvac	8	0		100%	63.06%–100%
MAL	6	0	—	100%	54.07%–100%	MAL	6	0		100%	54.07%–100%
NEG	17	0	—	100%	80.49%–100%	NEG	17	0		100%	80.49%–100%
Total control sera	103	5		**95.15%**	89.03%–98.41%	Total control sera	100	5		**95%**	88.72%–98.36%

*IgGcomp*											
**DENV TLb**	**23**	**23**	100%	—	85.18%–100%	DENV TLb	23	0	—	100%	85.18%–100%
**DENV END**	**55**	**55**	100%	—	93.51%–100%	DENV END	55	2	—	96.36%	87.47%–99.56%
ZIKV TLb	18	1	—	94.44%	73.97%–99.87%	**ZIKV**	**18**	**18**	100%	—	81.47%–100%
(ZIKV END)^a^	21	18	—	14.29%	3.05%–36.34%	**ZIKV END**	**21**	**21**	100%	—	83.89%–100%
NEG	17	0	—	100%	80.49%–100%	NEG	17	0	0	100%	80.49%–100%
Total control sera	35	1		**97.14%**	85.08%–99.93%	Total control sera	95	2		**97.89%**	92.60%–99.74%

Abbreviation: Confidence interval, CI, refers to the number of samples in the cohort. Bold entries represent sera groups which are infected with a to the antigen homologous virus and also total specificites of each test are highlighted in bold.

a Group was excluded from specificity measurements because of 90% DENV seroprevalence.
