# Supplementary material for: Specific detection of dengue and Zika virus antibodies using envelope proteins with mutations in the conserved fusion loop
Source: Emerg Microbes Infect. 2017 Nov 8;6(11):e99–. doi: 10.1038/emi.2017.87 (PMC5717088; doi:10.1038/emi.2017.87)
Supplement: Supplementary Table S1 [file emi201787x1.docx]

**Supplementary Table S1:** ROC positivity cut-offs of DENV- and ZIKV- Equad for IgG- and IgM- measurements. Cut-offs were calculated with negative sera (N=17) as controls to achieve optimal specificity and sensitivity

| **Detection** | **Antigen** | **ROC cut-off** | **Area under the ROC Curve (95% CI)** |
| --- | --- | --- | --- |
| **IgM** | DENV Equad | > 0.2732 | 0.9826 (0.9586 – 1.007) |
|  | ZIKV Equad | > 0.2734 | 0.9743 (0.9213 – 1.027) |
| **IgG** | DENV Equad | > 0.3323 | 1.0 (1.0) |
|  | ZIKV Equad | > 0.4425 | 1.0 (1.0) |
|  | DENV Equad + ZIKV comp | > 0.2899 | 1.0 (1.0) |
|  | ZIKV Equad + DENV comp | > 0.3437 | 1.0 (1.0) |

CI: confidence interval
