# Supplementary material for: Specific detection of dengue and Zika virus antibodies using envelope proteins with mutations in the conserved fusion loop
Source: Emerg Microbes Infect. 2017 Nov 8;6(11):e99–. doi: 10.1038/emi.2017.87 (PMC5717088; doi:10.1038/emi.2017.87)
Supplement: Supplementary Table S2 [file emi201787x2.docx]

**Supplementary Table S2:** Statistical analysis of IgM results with Sidak’s multiple comparison test (DENV Equad vs ZIKV Equad); asterisks indicate significant results (p-values are shown)

| **Group** | **Mean on DENV Equad** | **Mean on ZIKV Equad** | **Mean diff.** | **95% CI of diff.** | **Significant?** | **Summary** | **Adjusted P Value** |
| --- | --- | --- | --- | --- | --- | --- | --- |
| **DENV (n=54)** | 1.350 | 0.1580 | 1.192 | 0.9564 to 1.428 | Yes | **** | < 0.0001 |
| **ZIKV (n=16)** | 0.0943 | 0.7948 | -0.7005 | -1.133 to -0.2677 | Yes | *** | 0.0003 |
| **WNV (n=16)** | 0.1140 | 0.06178 | 0.05222 | -0.3806 to 0.4851 | No | ns | 0.9968 |
| **NEG (n=17)** | 0.0665 | 0.05121 | 0.01529 | -0.4046 to 0.4352 | No | ns | > 0.9999 |
