# Supplementary material for: Specific detection of dengue and Zika virus antibodies using envelope proteins with mutations in the conserved fusion loop
Source: Emerg Microbes Infect. 2017 Nov 8;6(11):e99–. doi: 10.1038/emi.2017.87 (PMC5717088; doi:10.1038/emi.2017.87)
Supplement: Supplementary Table S3 [file emi201787x3.docx]

**Supplementary Table S3:** Statistical analysis of IgG Results with Sidak’s multiple comparison test (DENV Equad vs ZIKV Equad); asterisks indicate significant results (p-values are shown)

| **Group** | **Mean on DENV Equad** | **Mean on ZIKV Equad** | **Mean diff.** | **95% CI of diff.** | **Significant?** | **Summary** | **Adjusted P Value** |
| --- | --- | --- | --- | --- | --- | --- | --- |
| **DENV TLa (n=9)** | 1.415 | 0.6747 | 0.7403 | 0.4246 to 1.056 | Yes | **** | < 0.0001 |
| **ZIKV TLa (n=12)** | 0.4730 | 1.479 | -1.006 | -1.279 to -0.7326 | Yes | **** | < 0.0001 |
| **WNV (n=24)** | 0.06401 | 0.02456 | 0.03945 | -0.1539 to 0.2328 | No | ns | 0.9995 |
| **TBEV (n=24)** | 0.08097 | 0.1575 | -0.07653 | -0.2699 to 0.1168 | No | ns | 0.9410 |
| **YFVvac (n=8)** | 0.03643 | 0.0350 | 0.001430 | -0.3334 to 0.3363 | No | ns | > 0.9999 |
| **JEV (n=4)** | 0.1130 | 0.1226 | -0.009600 | -0.4832 to 0.4640 | No | ns | > 0.9999 |
| **CHIKV (n=8)** | 0.08903 | 0.04823 | 0.0408 | -0.2941 to 0.3757 | No | ns | > 0.9999 |
| **Malaria (n=6)** | 0.06712 | 0.06261 | 0.00451 | -0.3822 to 0.3912 | No | ns | > 0.9999 |
| **NEG (n=17)** | 0.0548 | 0.04546 | 0.009340 | -0.2204 to 0.2391 | No | ns | > 0.9999 |
