# Supplementary material for: Specific detection of dengue and Zika virus antibodies using envelope proteins with mutations in the conserved fusion loop
Source: Emerg Microbes Infect. 2017 Nov 8;6(11):e99–. doi: 10.1038/emi.2017.87 (PMC5717088; doi:10.1038/emi.2017.87)
Supplement: Supplementary Table S4 [file emi201787x4.docx]

**Supplementary Table S4:** Statistical analysis of IgG competition results with Sidak’s multiple comparison test (DENV Equad vs ZIKV Equad); asterisks indicate significant results (p‑values are shown)

| **Group** | **Mean on DENV Equad** | **Mean on ZIKV Equad** | **Mean diff.** | **95% CI of diff.** | **Significant?** | **Summary** | **Adjusted P Value** |
| --- | --- | --- | --- | --- | --- | --- | --- |
| **DENV TLb (n=23)** | 2.124 | 0.1040 | 2.020 | 1.583 to 2.457 | Yes | **** | < 0.0001 |
| **DENV END (n=55)** | 2.294 | 0.1034 | 2.191 | 1.908 to 2.473 | Yes | **** | < 0.0001 |
| **ZIKV TLb (n=18)** | 0.1305 | 1.915 | -1.785 | -2.278 to -1.291 | Yes | **** | < 0.0001 |
| **ZIKV END (n=21)** | 2.257 | 1.797 | 0.4600 | 0.003091 to 0.9169 | Yes | * | 0.0476 |
| **NEG (n=17)** | 0.1196 | 0.1455 | -0.02590 | -0.5337 to 0.4819 | No | ns | > 0.9999 |
